# Evaluating the Association of Clinical Characteristics With Neutralizing Antibody Levels in Patients Who Have Recovered From Mild COVID-19 in Shanghai, China

**DOI:** 10.1001/jamainternmed.2020.4616

**Published:** 2020-08-18

**Authors:** Fan Wu, Mei Liu, Aojie Wang, Lu Lu, Qimin Wang, Chenjian Gu, Jun Chen, Yang Wu, Shuai Xia, Yun Ling, Yuling Zhang, Jingna Xun, Rong Zhang, Youhua Xie, Shibo Jiang, Tongyu Zhu, Hongzhou Lu, Yumei Wen, Jinghe Huang

**Affiliations:** 1 Shanghai Public Health Clinical Center and Key Laboratory of Medical Molecular Virology (MOE/NHC/CAMS), School of Basic Medical Sciences, Fudan University, Shanghai, China

## Abstract

**Importance:**

The coronavirus disease 2019 (COVID-19) pandemic caused by severe acute respiratory syndrome coronavirus 2 (SARS-CoV-2) threatens global public health. The association between clinical characteristics of the virus and neutralizing antibodies (NAbs) against this virus have not been well studied.

**Objective:**

To examine the association between clinical characteristics and levels of NAbs in patients who recovered from COVID-19.

**Design, Setting, and Participants:**

In this cohort study, a total of 175 patients with mild symptoms of COVID-19 who were hospitalized from January 24 to February 26, 2020, were followed up until March 16, 2020, at Shanghai Public Health Clinical Center, Shanghai, China.

**Exposures:**

SARS-CoV-2 infections were diagnosed and confirmed by reverse transcriptase–polymerase chain reaction testing of nasopharyngeal samples.

**Main Outcomes and Measures:**

The primary outcome was SARS-CoV-2–specific NAb titers. Secondary outcomes included spike-binding antibodies, cross-reactivity against SARS-associated CoV, kinetics of NAb development, and clinical information, including age, sex, disease duration, length of stay, lymphocyte counts, and blood C-reactive protein level.

**Results:**

Of the 175 patients with COVID-19, 93 were female (53%); the median age was 50 (interquartile range [IQR], 37-63) years. The median length of hospital stay was 16 (IQR, 13-21) days, and the median disease duration was 22 (IQR, 18-26) days. Variable levels of SARS-CoV-2–specific NAbs were observed at the time of discharge (50% inhibitory dose [ID50], 1076 [IQR, 448-2048]). There were 10 patients whose NAb titers were less than the detectable level of the assay (ID50, <40), and 2 patients who showed very high titers of NAbs, with ID50 levels of 15 989 and 21 567. NAbs were detected in patients from day 4 to 6 and reached peak levels from day 10 to 15 after disease onset. NAbs were unable to cross-react with SARS-associated CoV and NAb titers correlated with the spike-binding antibodies targeting S1 (*r* = 0.451; 95% CI, 0.320-0.564; *P* < .001), receptor binding domain (*r* = 0.484; 95% CI, 0.358-0.592; *P* < .001), and S2 regions (*r* = 0.346; 95% CI, 0.204-0.473; *P* < .001). NAb titers at the time of discharge were significantly higher in the 82 men (1417 [IQR, 541-2253]) than those in the 93 women (905 [IQR, 371-1687]) (median difference, 512; 95% CI, 82-688; *P* = .01) and at the time of follow-up in 56 male patients (1049 [IQR, 552-2454]) vs 61 female patients (751 [IQR, 216-1301]) (median difference, 298; 95% CI, 86-732; *P* = .009). Plasma NAb titers were significantly higher in 56 older (1537 [IQR, 877-2427) and 63 middle-aged (1291 [IQR, 504-2126]) patients than in 56 younger patients (459 [IQR, 225-998]) (older vs younger: median difference, 1078; 95% CI, 548-1287; *P* < .001; middle-aged vs younger: median difference, 832; 95% CI, 284-1013; *P* < .001). The NAb titers were correlated with plasma C-reactive protein levels (*r* = 0.508; 95% CI, 0.386-0.614; *P* < .001) and negatively correlated with lymphocyte counts (*r* = −0.427; 95% CI, −0.544 to −0.293; *P* < .001) at the time of admission.

**Conclusions and Relevance:**

In this cohort study, among 175 patients who recovered from mild COVID-19 in Shanghai, China, NAb titers to SARS-CoV-2 appeared to vary substantially. Further research is needed to understand the clinical implications of differing NAb titers for protection against future infection.

## Key Points

**Question:**

Are clinical characteristics of patients who recovered from mild coronavirus disease 2019 (COVID-19) associated with levels of neutralizing antibodies?

**Findings:**

In this cohort study of 175 patients who recovered from mild COVID-19, neutralizing antibody titers to severe acute respiratory syndrome coronavirus 2 (SARS-CoV-2) varied substantially at the time of discharge. In addition, neutralizing antibodies were not detected in 10 patients.

**Meaning:**

Further research is needed to understand the implications of variable levels of SARS-CoV-2–specific neutralizing antibodies for protection against future infections with SARS-CoV-2.

## Teaser

This cohort study examines the levels of neutralizing antibodies to severe acute respiratory syndrome coronavirus 2 in patients who recovered from mild coronavirus disease 2019.

## Introduction

As of July 30, 2020, severe acute respiratory syndrome coronavirus 2 (SARS-CoV-2) had caused a total of 16 812 755 infections and resulted in 662 095 deaths worldwide.^1^ In a study of 72 314 patients with SARS-CoV-2 infection in China, about 81% of the patients showed mild symptoms, but 14% had severe symptoms, such as dyspnea, high respiratory rate, and low blood oxygen saturation.^2^ Approximately 6.3% of patients died from respiratory or multiple organ failure.^1^ Currently, no licensed vaccine is available; there are limited drugs available for treatment (eg, remdesivir and dexamethasone), but most treatment is based on supportive care.

Neutralizing antibodies (NAbs) are important for viral clearance and are considered key to recovery and protection against viral diseases. The level of NAbs has been used as a standard to evaluate the efficacy of vaccines against smallpox, polio, and influenza viruses.^3^ Passive antibody therapy has been successfully used to treat infectious viral diseases, including those caused by SARS-associated CoV,^4^ influenza viruses,^5^ and Ebola virus.^6^ The efficacy of passive antibody therapy was associated with the concentration of NAbs in the plasma of recovered donors.^6^ Transfusion of convalescent plasma or serum from patients who have recovered from coronavirus disease 2019 (COVID-19) has been used for treatment and prophylaxis of infection with SARS-CoV-2.^7,8^

Consistent with other viral diseases, it has been assumed that patients who have recovered from COVID-19 would develop NAbs and may be protected from reinfection with SARS-CoV-2. To better understand the development of NAbs, we measured SARS-Cov-2–specific NAbs in plasma from patients with mild symptoms and examined the association between clinical characteristics and the level of NAbs.

## Methods

### Participants and Design

All patients admitted to Shanghai Public Health Clinical Center, Shanghai, China, from January 24 to February 26, 2020, who were diagnosed with laboratory-confirmed SARS-CoV-2 infection by positive results of reverse transcriptase–polymerase chain reaction testing of nasopharyngeal samples were isolated and hospitalized. We included in this study patients who were categorized as having mild symptoms according to the Guidelines on the Diagnosis and Treatment of Novel Coronavirus issued by the National Health Commission, China. Mild symptoms were defined as fever, respiratory symptoms, and radiologic evidence of pneumonia but not meeting any of the following manifestations: respiratory rate greater than 30/min, oxygen saturation levels less than 93%, ratio between arterial partial pressure of oxygen and fraction of inspired oxygen 300 mm Hg or less, or pulmonary imaging showing multilobular lesions or lesion progression exceeding 50% within 48 hours, as previously described.^9^ Patients with severe, critical COVID-19 were excluded from the study because they received passive antibody treatment before sample collection. Patients were discharged after meeting national treatment standards, including testing as afebrile for more than 3 days, improved respiratory symptoms, pulmonary imaging showing lessening of inflammation, and 2 sequential negative tests for nucleic acid in nasopharyngeal samples. Patients were followed up at 2 weeks post discharge until March 16, 2020. Healthy volunteers in Shanghai Public Health Clinical Center who did not have a history of exposure to SARS-CoV-2 and had negative tests on 2 occasions for SARS-CoV-2 viral RNA, were recruited as controls. This study was conducted under a clinical protocol approved by the investigational review board of Shanghai Public Health Clinical Center. All participants signed an informed consent form approved by the investigational review board; participants did not receive financial compensation. This study followed the Strengthening the Reporting of Observational Studies in Epidemiology (STROBE) reporting guideline for cohort studies.

Plasma was collected from the patients at the time of discharge. NAb titers were measured using a single-round pseudovirus infection assay. The reliability of single-round pseudovirus infection assay was validated by comparison with a viral cytopathology neutralization assay against live SARS-CoV-2 virus. Binding antibodies to SARS-CoV-2 spike (S) proteins, S1, receptor binding domain (RBD), and S2 were measured by enzyme-linked immunosorbent assay (ELISA). Plasma with high titers of NAbs was measured for cross-reactivity against SARS-associated CoV (SARS-CoV). To evaluate the kinetics of NAb development, sequential plasma samples were collected from admission to discharge at intervals of 2 to 4 days. NAb titers were also measured for patients who were followed up at 2 weeks post discharge and evaluated in pairwise comparison with NAb titers at the time of discharge. Clinical information, including age, sex, complete blood cell counts, blood biochemistry tests on admission, disease duration, and length of stay, were collected to explore the clinical characteristics associated with NAb levels.

### Materials and Assays

The human primary embryonic kidney cell line (293T) (CRL-3216), Vero E6 (CRL-1586) cells were obtained (American Type Culture Collection); 293T cells expressing human angiotensin-converting enzyme II (ACE2) (293 T/ACE2) were constructed as previously described^10^ and cultured in Dulbecco modified Eagle medium (DMEM) with fetal bovine serum (FBS), 10%. HEK293 cells expressed as SARS-CoV-2 S1, RBD, and S2 subunits, as well as SARS-CoV S1 and RBD subunits, were purchased (Sino Biological Company). The expression plasmids for SARS S protein pcDNA3.1-SARS-S (GenBank accession ABD72979.1) and SARS-CoV-2 S protein pcDNA3.1-SARS-CoV-2-S (GenBank accession NC_045512) were synthesized by Genscript. The vesicular stomatitis virus glycoprotein (VSV-G) envelope eukaryotic expression vector pHEF-VSVG and the HIV-1 Env-deficient luciferase reporter vector pNL4-3.Luc.R-E were obtained through the US National Institutes of Health AIDS Reagent Program.

Pseudovirus samples of SARS-CoV-2, SARS-CoV, and VSV-G virus were generated by cotransfection of 293T cells with pNL4-3.Luc.R-E-backbone and viral envelope protein expression plasmids, pcDNA3.1-SARS-CoV-2-S, pcDNA3.1-SARS-S, or pHEF-VSVG as previously described.^11^ The neutralization assay was performed in accordance with the following steps. First, 293 T/ACE2 cells were seeded in a 96-well plate at a concentration of 10^4^ cells per well in 100 μL of DMEM with FBS, 10%, and cultured for 12 hours. Then, 10 μL of heat-inactivated plasma was 5-fold serially diluted with DMEM with FBS, 10%, and mixed with 40 μL of pseudovirus. After incubation at 37 °C for 30 minutes, the mixture was added to cultured 293 T/ACE2 for infection. The culture medium was refreshed with 200 μL of DMEM with FBS, 10%, after 12 hours and incubated for an additional 48 hours. Assays were developed with a luciferase assay system (Promega), and the relative light units were read on a luminometer (Perkin Elmer, EnSight).

Viral cytopathology neutralization assay was performed in a biosafety level 3 facility in the School of Basic Medical Sciences, Fudan University. Briefly, the plasma samples were serially diluted using DMEM with FBS, 2%, and mixed with 200 plaque-formed units of SARS-CoV-2 SH01 isolate (GenBank accession MT121215.1). The mixtures were incubated at 37 °C for 1 hour before adding to 2 × 10^4^ Vero-E6 cells seeded in a 96-well plate. The cytopathologic changes of Vero-E6 cells was evaluated 3 days later and recorded by microscope.

### ELISA Analysis

For ELISA, SARS-CoV-2 RBD, S1, or S2 protein and SARS-CoV RBD or S1 protein (1 μg/mL) was coated on a 96-well plate (MaxiSorp Nunc-immuno, Thermo Scientific) and incubated overnight at 4 °C. Wells were blocked with nonfat milk, 5% (Biofroxx) in phosphate-buffered saline for 1 hour at room temperature, followed by incubation with 1:200, 1:400, or serially diluted heat-inactivated sera in disruption buffer (phosphate-buffered saline; FBS, 5%; bovine serum albumin, 2% BSA, and Tween-20, 1%) for 1 hour at room temperature. A 1:2500 dilution of horseradish peroxidase–conjugated goat antihuman IgG antibody (Jackson Immuno Research Laboratories) was added for 1 hour at room temperature. Wells were washed 5 times between each step with Tween-20, 0.2%, in phosphate-buffered saline. Wells were developed using ABTS (Thermo Scientific) for 30 minutes and read at 405 nm on a plate reader (Multiskan FC, Thermo Scientific).

### Outcomes

The primary outcome was the titers of SARS-CoV-2–specific NAbs, which was calculated as a 50% inhibitory dose (ID50) and expressed as the dilution of plasma that resulted in a 50% reduction of luciferase luminescence compared with virus control in single-round pseudovirus infection assay, with higher values indicating higher levels of NAbs. The NAb titers were defined as low (ID50, <500), medium-low (ID50, 500-999), medium-high (ID50, 1000-2500), and high (ID50, >2500), and the detection limit was 40. The secondary outcomes included spike-binding antibodies, which were expressed as absorbance (optical density [OD]) at 405 nm (OD 405) measured by ELISA, ranging from 0 to 5, with higher values indicating higher levels of binding antibodies; cross-reactivity against pseudotyped SARS-CoV virus; kinetics of NAbs development during disease duration; clinical characteristics, including age, sex, lymphocyte counts, blood C-reactive protein (CRP) level on admission, disease duration, and length of stay.

### Statistical Analysis

Statistical analyses were carried out using Prism, version 7.0 (GraphPad). The data are expressed as median (interquartile range [IQR]). All of the patients were included to analyze the NAb titers at time of discharge. The patients were numbered in the order of low to high ID50 values of NAbs. The nonparametric Mann-Whitney *t* test was used to compare the differences between 2 unpaired groups. The Kruskal-Wallis test was used to compare the differences between 3 or more groups and the Dunn multiple comparisons test was used to correct for multiple comparisons. Correlation coefficients with 95% CIs were calculated by the Spearman correlation coefficient test. The Wilcoxon matched-pairs signed-rank test was used to compare the NAbs difference between discharge and follow-up, with the exclusion of the patients who were lost to follow-up. All of the tests were 2-tailed, median difference with 95% CI was calculated, and *P* < .05 was considered statistically significant.

## Results

A total of 175 patients who recovered from COVID-19 and were discharged from the Shanghai Public Health Clinical Center as of February 26, 2020, were included in the study. Their symptoms were mild, and none of them was admitted to the intensive care unit. The median age of the patients was 50 (IQR, 37-63) years; 93 patients (53%) were women and 82 patients (47%) were men. The median length of hospital stay was 16 (IQR, 13-21) days, and the median disease duration was 22 (IQR, 18-26) days. A total of 117 of the 175 patients (67%) were followed up until March 16, 2020. Clinical information of all 175 patients is summarized in eTable 1 in the Supplement.

**Figure 1.**
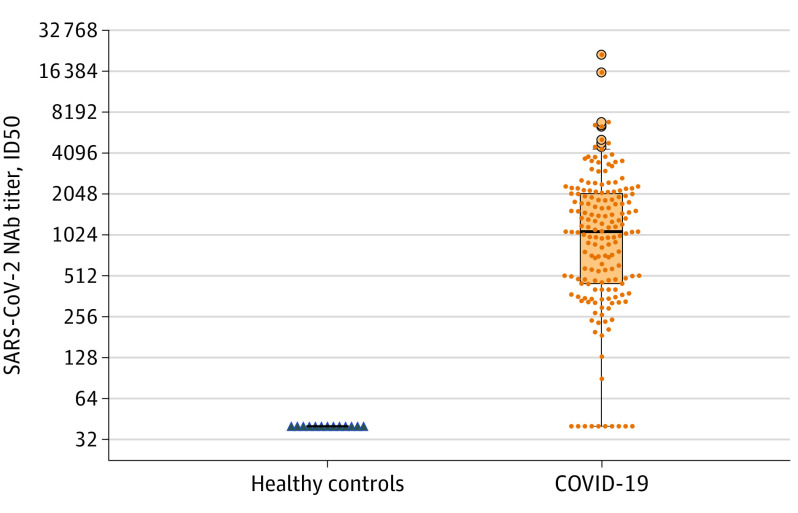
Neutralizing Antibody (NAb) Titers in Plasma From Patients Who Recovered From Coronavirus Disease 2019 (COVID-19) The 50% inhibitory dose (ID50) of severe acute respiratory syndrome coronavirus 2–specific (SARS-CoV-2) NAbs in plasma from 175 patients who recovered from COVID-19 (1076; interquartile range [IQR], 448-2048) were significantly higher than plasma from 13 healthy controls (40; IQR, 40-40); median difference, 1036; 95% CI, 534-1602; *P* < .001, Mann-Whitney test). The 10 patients who recovered without detectable NAbs are shown at the foot of the IQR bar.

Plasma samples were collected from patients who recovered from COVID-19 at the time of discharge and their neutralizing titer were measured against SARS-CoV-2 infection of 293T/ACE2 cells. As shown in eFigure 1A in the Supplement, plasma from the patients inhibited SARS-CoV-2 pseudovirus infection of 293T/ACE2 cells in a concentration-dependent manner. Most (165 of 175 [94%]) patients who recovered from COVID-19 developed significantly higher SARS-CoV-2–specific NAbs at the time of discharge compared with 13 uninfected controls (patients: 1076 [IQR, 448-2048] vs controls: 40 [IQR, 40-40]; median difference, 1036; 95% CI, 534-1602; *P* < .001) (Figure 1). The NAb titers in patients were variable, ranging from below the limit of detection (ID50, <40) to 21 567 at the time of discharge (eTable 1 in the Supplement). The reliability of the pseudovirus neutralization assay was validated using 3 plasma samples with different titers in patient 3 (ID50, <40), patient 170 (ID50, 5121), and patient 174 (ID50, 15 989), by the traditional viral cytopathology neutralization assay against live SARS-CoV-2 virus. Consistent with the pseudovirus neutralization results, plasma from patient 3 could not block live SARS-CoV-2 even at the lowest dilution (1:40), while plasma from patients 170 and 174 completely inhibited viral cytopathology at the dilutions of 1:320 and 1:1280, respectively (eFigure 1B in the Supplement).

Since SARS-CoV shares 77.2% amino acid identity with SARS-CoV-2 in their S proteins,^12^ the cross-reactivity of SARS-CoV-2 plasma in patients against SARS-CoV was evaluated. Plasma with high titers of NAbs showed higher binding abilities to the SARS-CoV-2 RBD, S1, and S2 domains (eFigure 1C in the Supplement). Moreover, plasma from these patients showed cross-binding to the SRAS-CoV RBD and S1 regions (eFigure 1D in the Supplement) but could not inhibit SARS-CoV in the pseudovirus neutralization assay. Twenty-six plasma samples from patients with COVID-19, which showed strong SARS-CoV-2–neutralizing activities, could neutralize neither SARS-CoV nor the control VSV-G (eFigure 1E in the Supplement).

**Figure 2.**
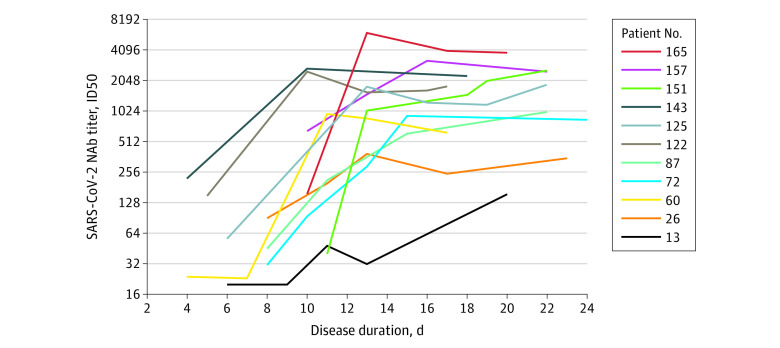
Kinetics of Neutralizing Antibody (NAb) Development During the Course of the Disease in 11 Patients Patients are numbered in order from low to high NAb titers at the time of discharge. Sequential plasma samples of the patients were collected from admission to discharge at 2- to 4-day intervals. The start time was set as symptom onset, which was determined according to admission presentation of the patients. Severe acute respiratory syndrome coronavirus 2 (SARS-CoV-2)–specific NAb titers (50% inhibitory dose [ID50]) at different time points post disease onset are shown.

Of the 11 patients for whom sequential plasma samples after admission were available, the kinetics of SARS-CoV-2–specific NAbs development were evaluated. NAb titers started to increase at days 4 to 6 post disease onset and reached their peak levels at days 10 to 15 post disease onset (Figure 2). The binding antibodies to the different domains (RBD, S1, and S2) of SARS-CoV-2 spike protein were also measured in these plasma samples. The kinetics of NAbs and binding antibodies targeting RBD, S1, and S2 domains were aligned for individual patients (eFigure 2A in the Supplement). The correlation of SARS-CoV-2–specific NAb titers and the spike-binding antibody levels were further evaluated in the plasma of the 175 recovered patients on the day of discharge. SARS-CoV-2–specific NAb titers correlated with spike-binding antibodies targeting RBD (*r* = 0.484; 95% CI, 0.358-0.592; *P* < .001), S1 (*r* = 0.451; 95% CI, 0.320-0.564; *P* < .001), and S2 (*r* = 0.346; 95% CI, 0.204-0.473; *P* < .001) (eFigure 2B in the Supplement). However, there were plasma samples from, for example, patients 3 and 8, that could not neutralize pseudovirus infection (ID50, <40) but developed high titers of spike-binding antibodies as measured by ELISA (eTable 1 and eFigure 2B in the Supplement).

**Figure 3.**
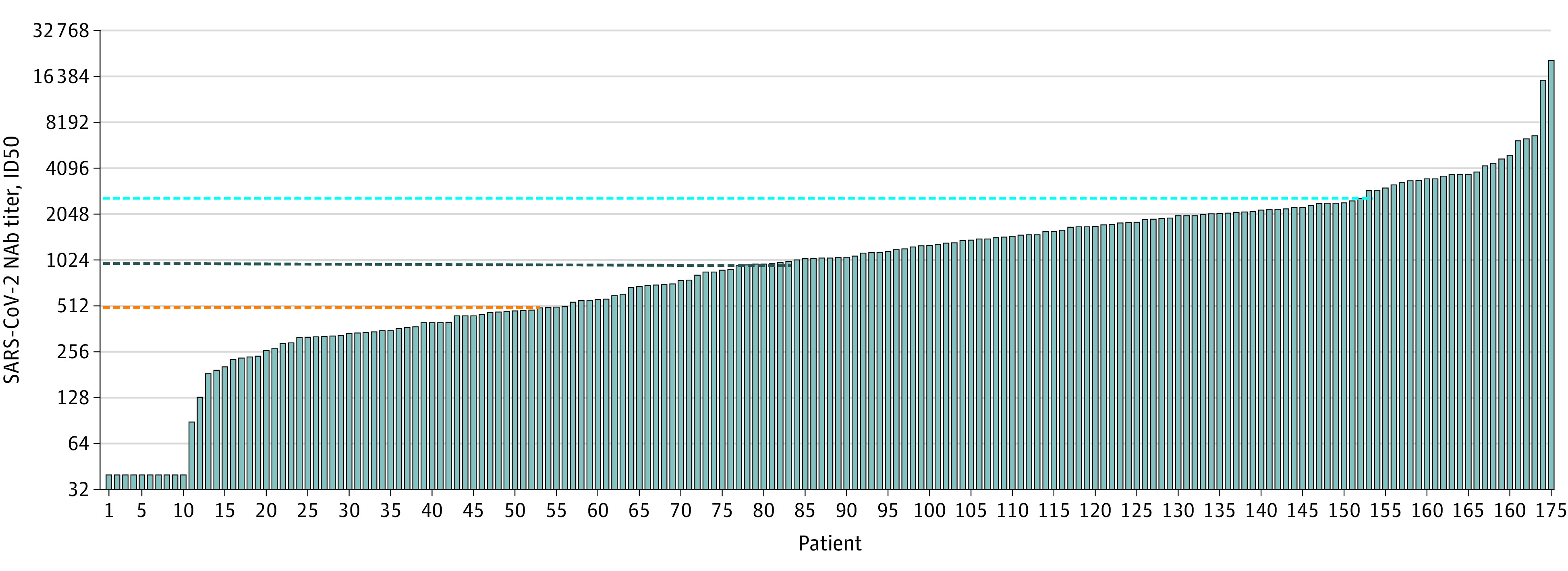
Variable Levels of Severe Acute Respiratory Syndrome Coronavirus 2 (SARS-CoV-2)-Specific Neutralizing Antibodies (NAbs) in Patients Who Recovered From Coronavirus Disease 2019 The SARS-CoV-2–specific NAb titer (50% inhibitory dose [ID50]) for each patient at the time of discharge is shown as an individual histogram. The dashed lines show the cutoff values of different NAb levels: low (ID50, <500), medium-low (ID50, 500-999), medium-high (ID50, 1000-2500), and high (ID50, >2500). Fifty-two patients (30%) had low levels (orange dashed line); 29 patients (17%) had medium-low levels, 69 patients (39%) had medium-high levels (dark blue dashed line), and 25 patients (14%) had high levels (bright blue dashed line).

The percentages of patients with different NAb titers are shown in Figure 3 and eTable 2 in the Supplement. Fifty-two patients (30%) who had recovered from COVID-19 generated low levels of NAbs (ID50, <500; median, 327; IQR, 189-404) (Figure 3; eTable 1 in the Supplement). NAb titers in 10 of these patients (19%) were below the limit of detection (ID50, <40), although SARS-CoV-2 was confirmed by polymerase chain reaction in all of these patients (eTable 1 in the Supplement). Those 10 patients who did not develop NAbs were younger (median age, 34 [IQR, 29-39.25] years) and most were women (8 [80%]) (eTable 1 in the Supplement). NAb titers were medium-low in 29 patients (17%) (ID50, 500-999; median, 715 [IQR, 571-881]), medium-high in 69 patients (39%) (ID50, 1000-2500; median, 1642 [IQR, 1282-2090]), and high in 25 patients (14%) (ID50, >2500; median, 3800 [IQR, 3316-4970]) (Figure 3; eTable 2 in the Supplement). The patients in this cohort who developed high titers of NAbs (>2500) were older (median age, 63 [IQR, 44-68] years) and 14 were men (56%) (eTable 1 in the Supplement). The NAb titers in 82 men (47%) (1417 [IQR, 541-2253]) were significantly higher than those in 93 women (53%) at the time of discharge (905 [IQR, 371-1687]; median difference, 512; 95% CI, 82-688; *P* = .01) (eFigure 3A in the Supplement).

In the 117 patients available for follow-up at 2 weeks post discharge, the median NAb titer in plasma at follow-up was 886 (IQR, 378-1658), which was significantly lower than that at the time of discharge (1110 [IQR, 447-2042]; median difference, –224; 95% CI, –241 to –21; *P* < .01). Furthermore, the patients who did not generate NAbs at the time of discharge did not develop detectable NAbs at the time of follow-up (eTable 1 in the Supplement). NAb titers in 39 patients (33%) at follow-up were below 500 (median, 212 [IQR, 144-379]) (eTable 2 in the Supplement). Among the 117 patients, NAb titers in 56 men (48%) (1049 [IQR, 522-2454]) were still significantly higher than those in 61 women (52%) (751 [IQR, 216-1301]; median difference, 298; 95% CI, 86-732; *P* = .009) (eFigure 3B in the Supplement).

We further explored the clinical manifestations associated with the NAb levels of the patients who recovered from COVID-19. We found that older patients developed higher titers of NAbs than younger patients. The 175 patients were divided into 3 groups based on their age: younger (15-39 years, n = 56), middle-aged (40-59 years, n = 63), and older (60-85 years, n = 56). At the time of discharge, NAb titers of the older (1537 [IQR, 877-2427]) and middle-aged (1291 [IQR, 504-2126]) patients were significantly higher than those of the younger patients (younger: 459 [IQR, 225-998]; median difference, 1078; 95% CI, 548-1287; *P* < .001 vs younger: median difference, 832; 95% CI, 284-1013; *P* < .001) (eFigure 4A; eTable 3 in the Supplement). A moderate correlation was observed between age and NAb titers (*r* = 0.414; 95% CI, 0.279-0.533; *P* < .001) (eFigure 4B in the Supplement). Older and middle-aged patients also had significantly higher levels of spike-binding antibodies than those of younger patients in ELISA assay in either targeting RBD (older: OD 405, 1.995 [IQR, 1.365-2.8]; median difference, 0.885; 95% CI, 0.31-1.01; *P* < .001 and middle-aged: OD 405, 1.66 [IQR, 1.04-2.32]; median difference, 0.52; 95% CI, 0.03-0.7; *P* = .03 vs younger: OD 405, 1.14 [IQR, 0.783-2.05]), S1 (older: OD 405, 1.44 [IQR, 0.872-1.92]; median difference, 0.645; 95% CI, 0.23-0.75; *P* < .001 and middle-aged: OD 405, 1.2 [IQR, 0.87-1.77]; median difference, 0.405; 95% CI, 0.13-0.58; *P* = .002 vs younger: OD 405, 0.795 [IQR, 0.592-1.258]), or S2 (older: OD 405, 2.44 [IQR, 1.278-3.473]; median difference, 0.93; 95% CI, 0.23-1.21; *P* = .002 and middle-aged: OD 405, 2.01 [IQR, 1.4-2.72]; median difference, 0.5; 95% CI, 0.12-0.82; *P* = .006 vs younger: OD 405, 1.51 [IQR, 1.008-2.178]) (eFigure 4C in the Supplement).

It has been reported that older patients with COVID-19 are at higher risk of developing severe and critical disease than younger adults.^13^ Low lymphocyte counts and high CRP levels were usually associated with poor outcome among patients with COVID-19 .^14^ Consistent with the previous reports, the older and middle-aged patients in this cohort had significantly lower lymphocyte counts (*r* = −0.355; 95% CI, −0.482 to −0.214; *P* < .001) (eFigure 5A in the Supplement) and higher CRP levels (*r* = 0.439; 95% CI, 0.307-0.554; *P* < .001) (eFigure 5B in the Supplement) than younger patients at the time of admission. NAb titers at discharge negatively correlated with blood lymphocyte counts at admission (*r* = −0.427; 95% CI, −0.544 to −0.293; *P*<.001) (eFigure 5C in the Supplement) but positively correlated with blood CRP levels at admission (*r* = 0.508; 95% CI, 0.386-0.614; *P* < .001) (eFigure 5D in the Supplement).

## Discussion

In this observational study, NAbs in 175 patients who recovered from mild COVID-19 were evaluated by pseudovirus neutralization assay. The titers of SARS-COV-2–specific NAbs varied substantially, including 10 patients in whom NAbs were below the limit of detection.

Most patients who recovered from mild COVID-19 developed SARS-CoV-2–specific NAbs at the convalescent phase of infection. The titers of NAbs reached their peak at 10 to 15 days after disease onset. Antibodies targeting different domains of S protein, including S1, RBD, and S2, may all contribute to the neutralization. Plasma from patients who recovered from mild COVID-19 showed cross-binding but did not neutralize SARS-CoV, suggesting that the antigenicity of SARS-CoV-2 is distinct from that of SARS-CoV. Conserved epitopes may exist between SARS-CoV-2 and SARS-CoV since they share 77.2% identical amino acids in their spike proteins.^12^ Few reports have reported that SARS-CoV–specific monoclonal NAbs could cross-neutralize SARS-CoV-2 pseudovirus infection.^15,16,17^ Findings noted in this study suggest that cross-neutralizing antibodies targeting the conserved epitopes of SARS-CoV and SARS-CoV-2 may not be easily elicited during SARS-CoV-2 infection.

We noted variable levels of NAbs in patients. Thirty percent of the patients developed NAbs with titers less than 500 after COVID-19, and 10 patients had NAb titers under the detectable limit of the assay (ID50, <40). However, the disease duration of these 10 patients was not significantly different compared with the duration in the other patients. It is not clear how these patients recovered without developing detectable virus-specific NAbs. Whether other immune responses, including T cells or cytokines, contributed to the recovery of these patients and whether these patients are at risk for reinfection is not known. Two patients had very high titers of Nabs (ID50, 15 989 and 21 567). Studies on how these patients developed high titers of NAbs may provide useful information for the development of SARS-CoV-2 vaccines. In addition, the variability in NAb titers demonstrates the importance of titrating convalescent plasma before its use for prevention and treatment of COVID-19.

We found the NAb titers in patients appeared to be associated with age. Older patients had significantly higher titers of NAbs than younger patients in this cohort. Age has been reported to be an important predictor of adverse disease outcomes after infection with coronavirus, including SARS-CoV,^18^ Middle East respiratory syndrome-CoV,^19^ and SARS-CoV-2.^13^ Previous studies in SARS-CoV–infected macaques revealed that aged macaques induced an elevated innate immune response, resulting in more severe pathologic changes than in younger adult macaques.^20^ The older patients in this cohort also had higher blood CRP levels and lower lymphocyte counts at the time of admission; the higher blood CRP levels suggests induction of a stronger innate immune response than in younger patients. Furthermore, NAb titers at discharge positively correlated with blood CRP levels but negatively correlated with lymphocyte counts at admission, suggesting that high levels of NAbs may be a consequence of strong inflammation or innate immune response in these older patients in whom the lower lymphocyte count may reflect poorer T cell responses. Older patients developed higher NAb titers yet tend to have worse outcomes from COVID-19. This finding calls into question whether SARS-CoV-2 NAbs play protective roles in illness as assumed.

### Limitations

This study has several limitations. First, the kinetics of NAb development were based on 11 of the 175 patients owing to the limited availability of sequential samples. Second, the patients were followed up for 2 weeks after discharge and only 117 patients were available for follow-up. Third, the disease duration was calculated from disease onset to discharge, which was longer than the symptom duration. Fourth, patients in severe and critical condition were excluded from the study because they received passive antibody treatment before sample collection.

## Conclusions

The findings of this study noted that, among patients who recovered from mild COVID-19 in Shanghai, China, neutralizing antibody titers to SARS-CoV-2 appeared to vary substantially. The potential clinical implications of these findings for vaccine development and future protection from infection are unknown.

## Notes

Supplement. eTable 1. Clinical Characteristics of Patients Who Recovered From COVID-19 eTable 2. Clinical Characteristics of COVID-19 Recovered Patients With Low, Medium-Low, Medium-High, and High Titers of SARS-CoV-2–Specific NAbs on Day of Discharge and Revisit eTable 3. Clinical Characteristics and SARS-CoV-2–Specific NAb Titers of Younger, Middle-Aged, and Older COVID-19 Recovered Patients eFigure 1. Plasma From Patients Who Recovered From COVID-19 Specifically Inhibited SARS-CoV-2 Infection but not SARS-CoV eFigure 2. Correlation between SARS-CoV-2 NAb titers and Spike-Binding Antibodies eFigure 3. Male Patients Who Recovered From COVID-19 Developed Significantly Higher NAb Titers Than Females eFigure 4. Older and Middle-Aged Patients Who Recovered From COVID-19 Developed Higher Levels of SARS-CoV-2–Specific NAbs Than Younger Recovered Patients eFigure 5. Correlation Between SARS-CoV-2–Specific NAb Titers at Discharge and Lymphocyte Count and CRP Levels at Admission
