# Supplementary material for: Evaluating the Association of Clinical Characteristics With Neutralizing Antibody Levels in Patients Who Have Recovered From Mild COVID-19 in Shanghai, China
Source: JAMA Intern Med. 2020 Aug 18;180(10):1356–62. doi: 10.1001/jamainternmed.2020.4616 (PMC9377417; doi:10.1001/jamainternmed.2020.4616)
Supplement: Supplement. — eTable 1. Clinical Characteristics of Patients Who Recovered From COVID-19 eTable 2. Clinical Characteristics of COVID-19 Recovered Patients With Low, Medium-Low, Medium-High, and High Titers of SARS-CoV-2–Specific NAbs on Day of Discharge and Revisit eTable 3. Clinical Characteristics and SARS-CoV-2–Specific NAb Titers of Younger, Middle-Aged, and Older COVID-19 Recovered Patients eFigure 1. Plasma From Patients Who Recovered From COVID-19 Specifically Inhibited SARS-CoV-2 Infection but not SARS-CoV eFigure 2. Correlation between SARS-CoV-2 NAb titers and Spike-Binding Antibodies eFigure 3. Male Patients Who Recovered From COVID-19 Developed Significantly Higher NAb Titers Than Females eFigure 4. Older and Middle-Aged Patients Who Recovered From COVID-19 Developed Higher Levels of SARS-CoV-2–Specific NAbs Than Younger Recovered Patients eFigure 5. Correlation Between SARS-CoV-2–Specific NAb Titers at Discharge and Lymphocyte Count and CRP Levels at Admission [file jamainternmed-e204616-s001.pdf]

Supplementary Online Content

Wu F, Liu M, Wang A, et al. Evaluating the association of clinical characteristics with neutralizing antibody levels in patients who have recovered from mild COVID-19 in Shanghai, China. *JAMA Intern Med*. Published online August 18, 2020. doi:10.1001/jamainternmed.2020.4616

eTable 1. Clinical Characteristics of Patients Who Recovered From COVID-19

eTable 2. Clinical Characteristics of COVID-19 Recovered Patients With Low, Medium-Low, Medium-High, and High Titers of SARS-CoV-2–Specific NAbs on Day of Discharge and Revisit

eTable 3. Clinical Characteristics and SARS-CoV-2–Specific NAb Titers of Younger, Middle-Aged, and Older COVID-19 Recovered Patients

eFigure 1. Plasma From Patients Who Recovered From COVID-19 Specifically Inhibited SARS-CoV-2 Infection but not SARS-CoV

eFigure 2. Correlation between SARS-CoV-2 NAb titers and Spike-Binding Antibodies

eFigure 3. Male Patients Who Recovered From COVID-19 Developed Significantly Higher NAb Titers Than Females

eFigure 4. Older and Middle-Aged Patients Who Recovered From COVID-19 Developed Higher Levels of SARS-CoV-2–Specific NAbs Than Younger Recovered Patients

eFigure 5. Correlation Between SARS-CoV-2–Specific NAb Titers at Discharge and Lymphocyte Count and CRP Levels at Admission

This supplementary material has been provided by the authors to give readers additional information about their work.

eTable 1. Clinical Characteristics of Patients Who Recovered From COVID-19

Patient	Sex	Age, y	Temperature, °C	Symptoms	TLC, /µL[a]	CRP level, mg/dL[b]	Duration, d		NAb titer, ID50		Binding, OD 405[d]		
							Hospital	Disease[c]	Discharge	Follow-up	RBD	S1	S2
1	F	30s	37.8	Fever, nasal congestion	2020	<0.050	22	31	40	NA	1.07	1.02	1.13
2	F	30s	37.6	Cough, muscle aches, nasal congestion	1020	0.616	17	22	40	40	0.22	0.23	0.29
3	M	10s	37.7	Nasal congestion, cough	2450	0.630	9	12	40	40	2.96	2.45	2.81
4	F	30s	38.1	Cough	1300	0.560	8	12	40	40	0.55	0.46	0.42
5	M	40s	37.9	Cough, chest pain	1590	<0.050	13	14	40	40	1.16	0.87	1.04
6	F	30s	37.4	Fatigue	1420	<0.050	13	15	40	NA	0.70	0.67	0.63
7	F	60s	37.2	Chills	1830	0.251	18	22	40	40	0.35	0.57	0.42
8	F	30s	38.1	Sore throat, cough, chest tightness	1350	0.398	21	23	40	40	3.46	2.37	3.28
9	F	20s	38	Cough	2690	<0.050	8	9	40	NA	0.53	0.45	0.52
10	F	30s	38.4	Cough, dizziness	2560	<0.050	12	23	40	40	0.36	0.47	0.65
11	M	30s	37	Fever	1750	<0.050	8	9	89	232	3.24	2.08	3.06
12	F	20s	37.6	Fever	2860	<0.050	12	16	130	391	0.31	0.21	0.28
13	M	40s	37.8	Cough	1420	0.088	9	20	186	165	0.51	0.54	1.36
14	F	40s	37.2	Cough, nasal congestion, fatigue	1060	0.225	15	26	196	NA	0.51	0.35	0.75
15	F	30s	37	Fever, sore throat	1960	<0.050	15	24	206	167	0.89	0.80	0.81
16	M	30s	37.6	Fever, sore throat	1170	<0.050	26	30	230	NA	1.48	1.22	1.81
17	F	60s	37.5	Cough, nasal congestion	1460	0.354	16	24	235	81	2.69	1.78	2.69
18	F	30s	38.5	Chills, headache	630	<0.050	18	22	239	105	0.43	0.26	1.59
19	F	20s	37.9	Fever, cough	1270	0.344	16	21	242	NA	1.34	1.11	1.30

20	F	40s	36.8	Fever	1750	1.490	17	18	264	NA	0.78	0.74	0.99
21	M	50s	38.3	Cough	980	1.028	13	17	273	464	0.82	0.76	1.56
22	M	30s	36	Fever, cough	1890	0.120	8	17	294	NA	2.29	1.37	1.94
23	F	40s	39.2	Fever, cough, sputum	520	1.268	29	31	297	NA	2.87	1.94	4.61
24	M	20s	38.1	Fever	1420	1.773	13	16	322	NA	0.82	0.59	0.98
25	F	30s	38.1	Fever, cough	730	<0.050	23	25	324	220	1.68	0.63	1.63
26	M	30s	38.2	Fever, cough	2560	<0.050	17	23	326	200	0.79	0.78	0.81
27	M	30s	38.4	Fever, cough	2350	0.697	13	21	327	292	0.94	0.65	0.86
28	F	20s	39.7	Fever	1080	0.746	14	18	330	NA	2.28	0.98	2.26
29	F	50s	38	Chills, nasal congestion, cough	650	1.117	9	15	333	453	0.91	1.06	1.11
30	F	60s	38.1	Fever, cough, sputum	900	3.461	15	17	343	435	1.52	0.93	2.70
31	M	30s	37.7	Soreness, headache, nasal congestion	1030	0.064	14	20	344	NA	0.77	0.71	0.92
32	M	30s	38	Fever	650	0.239	15	20	346	822	0.99	0.37	1.48
33	F	50s	37.6	Fever, cough, sputum	1720	2.859	14	19	350	116	1.64	0.86	2.01
34	M	20s	37.2	Nasal congestion, itchy throat, headache	1550	0.102	20	21	356	NA	0.57	0.65	1.07
35	F	60s	37.8	Fever, sore throat, headache	1400	<0.050	24	29	357	377	2.15	1.53	2.56
36	F	60s	37.5	Fever, cough	750	0.460	18	23	369	462	1.45	1.05	1.14
37	F	20s	37.6	Fever, cough	1790	0.058	14	18	373	141	1.72	1.18	1.04
38	F	30s	38	Fever, cough	1900	0.249	14	20	378	115	1.84	0.79	2.06
39	F	30s	38	Fever, headache, cough, nasal congestion	1040	1.600	17	19	403	213	0.83	1.00	1.47
40	M	30s	36.4	Cough	1390	0.875	9	13	404	1052	1.07	0.66	1.32
41	F	60s	37.4	Fever	1070	0.407	22	29	404	1092	1.35	0.96	0.62
42	F	50s	38.5	Fever	1880	<0.050	27	29	405	NA	1.24	0.88	2.25
43	F	40s	38	Dizziness, fatigue	640	0.135	11	13	446	142	1.22	1.07	1.30
44	M	30s	37.7	Fever, fatigue	1490	0.947	12	14	448	138	0.52	0.25	1.52

45	F	40s	38	Nasal congestion, headache	1530	0.606	11	14	448	1054	1.00	0.60	2.22
46	F	60s	38.3	Fever, fatigue, cough	1280	3.693	29	33	457	1557	2.02	1.26	4.25
47	F	30s	38.2	Fever, fatigue, chest tightness	1080	0.794	30	33	471	NA	2.38	1.13	1.44
48	M	30s	38.3	Fever, cough	870	1.226	24	27	473	379	2.38	1.73	2.09
49	M	50s	37.6	Fever, cough	1000	1.046	8	19	480	519	0.58	0.34	0.66
50	F	50s	37.2	Chest tightness	1070	<0.050	13	15	481	262	0.75	0.59	0.92
51	M	50s	38.5	Fever	1570	6.257	25	29	484	NA	2.38	1.77	1.93
52	M	50s	37.7	Fever, cough	910	1.877	11	21	488	NA	2.71	2.11	3.25
53	F	40s	38	Fever	1500	<0.050	16	27	504	270	0.72	0.53	1.93
54	F	70s	37.4	Abdominal distention, nausea	720	2.768	25	31	507	1233	2.81	2.22	3.45
55	M	30s	37.4	Cough	1190	0.080	12	15	512	453	0.21	0.14	1.00
56	F	60s	37.8	Fever, cough	2510	1.351	17	21	513	181	1.41	0.96	1.47
57	M	50s	37.4	Cough	980	<0.050	11	18	551	1453	1.58	1.06	2.05
58	F	60s	36.8	Cough	1320	0.487	17	22	562	NA	1.14	0.61	1.81
59	M	40s	37.1	Chest tightness, fatigue	1750	0.959	21	23	568	266	0.44	0.26	1.98
60	F	60s	37.8	Fever, cough	1830	<0.050	14	17	574	186	0.57	0.52	0.95
61	M	20s	38.2	Fever, dizziness	2260	<0.050	24	27	576	NA	0.82	0.65	1.28
62	M	40s	37.2	Fever	1430	0.786	15	16	607	837	0.52	0.29	1.89
63	F	50s	38.5	Fever	1570	0.746	28	31	623	NA	0.91	0.62	1.34
64	M	60s	37.2	Cough	1070	0.624	27	29	689	NA	0.57	0.44	1.62
65	M	30s	37.6	Fever	1590	0.867	16	23	697	803	1.78	1.27	1.92
66	F	30s	37	Fever	2000	0.329	15	16	710	NA	1.81	1.31	2.68
67	M	50s	37.2	Cough, expectoration	750	1.238	7	14	715	285	1.52	1.33	2.89
68	F	30s	38.5	Nasal congestion, cough	1870	<0.050	20	26	718	NA	2.42	1.71	3.13
69	F	20s	36.8	Dizziness, nasal congestion	1470	<0.050	12	14	725	NA	1.66	0.25	0.83

70	F	60s	36.8	Fever	740	1.139	13	15	765	408	0.75	0.42	1.00
71	F	30s	37.8	Fever	1350	1.387	22	31	768	NA	2.21	1.52	4.01
72	F	30s	37.3	Fever	1250	0.693	13	24	827	NA	1.58	0.86	2.19
73	F	70s	38	Fever	1450	2.144	14	19	868	NA	2.45	1.69	4.72
74	M	40s	37.4	Cough	590	7.635	8	17	870	1075	1.61	1.25	2.74
75	M	20s	37.3	Chills, fatigue, cough, expectoration	1810	<0.050	13	15	892	1732	1.06	0.89	2.62
76	F	60s	37.5	Cough, chills, fever, muscle ache	610	2.211	14	19	905	NA	1.47	0.63	3.97
77	M	30s	37.7	Fever, muscle ache	1680	0.792	16	19	964	938	0.78	0.52	2.00
78	M	60s	37.3	Fever, cough	1520	<0.050	22	26	972	NA	1.86	1.86	3.09
79	F	30s	37.7	Fever, sore throat, fatigue	2490	0.442	14	18	979	219	1.23	0.92	2.11
80	M	30s	37.4	Chills, cough	1790	<0.050	17	29	979	NA	0.87	0.60	3.26
81	F	60s	38.3	Cough, chills, nausea	1140	0.625	12	24	989	1137	1.49	1.03	3.30
82	M	30s	38.9	Fever	1090	0.819	8	16	1004	727	1.06	0.81	1.86
83	F	30s	37.7	Fever	1230	0.186	14	24	1022	532	0.80	0.60	2.14
84	F	40s	38.1	Headache, muscle ache, fatigue	720	0.777	23	27	1046	1982	0.79	0.57	2.50
85	M	60s	36.5	Fever	1370	<0.050	20	20	1066	NA	1.01	0.59	1.41
86	F	50s	37.4	Shortness of breath	1640	0.161	16	18	1070	NA	1.04	0.46	1.58
87	F	50s	39	Fever, fatigue, dizziness	1200	0.357	18	22	1075	834	1.46	1.20	2.37
88	F	50s	38.3	Fever, cough	690	1.150	25	26	1076	1373	2.48	2.24	2.64
89	M	40s	37.5	Fever	1110	<0.050	18	20	1081	1044	0.50	0.31	1.33
90	F	40s	38.5	Fever, headache, chest tightness	510	2.920	21	25	1088	728	1.66	1.07	2.68
91	F	60s	38.4	Fever, fatigue, muscle ache	1050	1.094	10	16	1110	509	2.76	2.14	2.02
92	M	50s	38.4	Chills, dry cough, poor appetite	920	3.575	15	16	1158	1045	3.29	2.33	4.32
93	F	60s	37.3	Fever, chills, nausea	850	1.898	12	17	1163	424	1.08	0.86	1.89
94	M	60s	38.3	Cough	1740	5.475	15	17	1171	NA	1.44	0.96	1.49

95	M	40s	38.1	Fever	920	4.410	18	22	1186	1268	2.03	1.33	1.57
96	F	40s	38.2	Fever, chest tightness	1380	0.794	15	19	1219	NA	2.06	1.57	4.13
97	F	60s	37.8	Fever, fatigue	1110	3.138	16	24	1232	886	1.63	1.49	1.26
98	F	60s	37.9	Fever, cough, chest tightness	840	0.156	7	26	1272	996	3.08	2.12	4.50
99	M	40s	37.5	Fever, chest tightness	770	1.646	16	24	1291	629	2.20	1.27	3.15
100	F	60s	36.5	Fever	1250	0.254	16	26	1303	NA	1.31	0.78	3.29
101	M	60s	38.6	Fever, fatigue, muscle ache	1110	0.457	14	20	1323	532	0.99	0.50	1.25
102	F	30s	38.1	Fever, cough	620	1.314	25	30	1348	NA	1.21	1.28	0.74
103	F	40s	38.7	Fever, cough, chills	990	5.214	10	20	1359	1026	3.29	2.17	1.31
104	M	50s	37.5	Fever, body aches	1490	1.332	17	21	1401	1487	1.70	1.52	2.93
105	F	70s	38	Fever, cough	1010	0.467	12	20	1411	2004	0.57	0.34	0.76
106	M	70s	39	Fever, muscle ache	940	7.904	12	16	1433	866	2.41	1.42	4.63
107	F	30s	38.3	Cough, expectoration, fatigue, chills	1660	1.708	23	32	1435	NA	2.73	2.91	3.28
108	F	60s	37.2	Fever, cough	1320	<0.050	22	23	1462	539	1.23	0.86	1.03
109	M	40s	39	Fever, cough	940	0.986	29	34	1482	NA	1.42	1.00	0.85
110	M	30s	37.5	Nasal congestion, fever	2060	1.416	13	17	1496	NA	0.88	0.67	1.80
111	M	30s	37.6	Fever, fatigue	960	0.518	17	23	1522	955	0.70	0.46	1.05
112	F	50s	38.7	Fever, fatigue, cough	570	0.195	13	18	1531	1368	4.15	2.52	4.52
113	M	50s	39.1	Fever, fatigue, cough	510	2.518	19	22	1531	2278	1.96	1.49	2.38
114	F	50s	37.6	Fever	1300	0.103	7	21	1605	1177	1.46	0.87	1.35
115	F	60s	36.8	Fever, nausea	910	<0.050	28	29	1611	NA	1.41	1.59	0.81
116	F	50s	39	Fever, dry cough	2200	3.015	30	32	1642	3922	2.16	2.24	1.95
117	M	50s	37.5	Fatigue, chest tightness, muscle ache	1480	1.384	22	23	1715	NA	1.76	1.60	1.53
118	F	40s	37.1	Cough	1130	0.702	14	17	1731	1599	1.42	1.28	1.40
119	F	60s	38.5	Fever, cough	670	2.581	17	24	1732	1773	3.57	3.06	4.11

120	M	50s	38.6	Fever, dry cough, sore throat	930	2.491	27	31	1735	1648	2.32	3.63	1.53
121	M	50s	38.6	Fever	900	2.656	27	29	1775	NA	1.88	1.53	2.78
122	F	60s	38.6	Fever	1140	4.222	14	17	1786	2140	2.53	1.72	2.69
123	F	70s	37	Cough	930	2.265	12	15	1832	1019	2.42	1.94	0.54
124	M	50s	38.4	Fever, cough	870	2.358	13	18	1842	3196	2.09	1.24	1.70
125	M	60s	38	Fever	1600	7.010	17	22	1853	733	2.37	1.63	2.91
126	M	70s	38	Fever	520	7.615	20	22	1931	1667	2.96	2.41	4.73
127	M	60s	37.2	Fever, cough	132	0.271	26	29	1940	3477	3.01	2.66	2.96
128	M	50s	36.9	Intermittent fever	1160	1.489	16	23	1966	437	1.11	1.15	1.20
129	M	30s	37.8	Fever, cough	800	0.664	14	24	1978	666	2.12	1.44	2.23
130	F	20s	37.6	Fever, cough	720	0.058	14	17	2039	1153	0.77	0.59	1.03
131	F	30s	38.5	Fever	790	0.262	20	21	2045	782	2.54	1.63	1.32
132	F	70s	37.5	Fever, chills, cough	1390	0.672	25	28	2048	NA	3.09	2.21	2.32
133	F	50s	37.6	Cough	910	3.133	13	15	2080	NA	2.86	2.14	0.62
134	M	40s	37.5	Fever, chills, cough	1810	0.865	8	11	2100	1196	1.53	0.91	3.28
135	M	70s	38.4	Fever	1120	1.790	17	19	2114	NA	0.94	0.33	3.48
136	M	40s	38.1	Fever, cough	710	4.175	10	16	2126	NA	3.70	1.39	2.27
137	M	60s	37.3	Cough, expectoration	950	7.603	11	18	2155	2829	1.97	4.49	3.43
138	M	40s	38	Chest tightness, shortness of breath	910	3.467	8	22	2161	NA	2.62	2.46	4.35
139	F	60s	38	Fever, muscle ache	1790	1.088	17	22	2175	NA	3.07	1.57	1.18
140	F	70s	36.8	Sore throat, poor appetite	1470	0.322	29	31	2232	NA	4.66	3.46	4.70
141	M	30s	38.1	Fever	1720	1.622	17	19	2241	729	1.41	0.64	1.50
142	M	60s	38	Fever, fatigue, poor appetite, cough	440	3.196	10	19	2250	NA	2.52	1.16	4.49
143	M	60s	38	Fever	910	4.646	16	18	2262	1456	2.06	1.01	2.13
144	M	50s	37.8	Headache, cough, arthralgia	700	4.181	11	16	2323	4970	1.77	1.27	1.75

145	M	20s	38	Fever	2250	0.358	10	22	2324	NA	1.82	1.02	1.39
146	M	40s	38.3	Fever	1370	1.436	22	26	2389	1370	2.05	1.17	4.12
147	M	20s	38.2	Fever, nausea	770	0.526	23	27	2454	2513	1.30	0.67	3.21
148	F	40s	38.8	Fever, cough, nausea	900	0.512	16	23	2466	751	1.58	1.84	1.52
149	F	50s	38.7	Cough, chest tightness, fatigue	850	4.132	21	25	2474	1868	2.32	1.11	2.14
150	M	60s	37.8	Fever, cough	580	4.333	10	15	2482	NA	1.65	0.86	3.68
151	F	30s	38	Chills, fatigue	890	2.582	13	22	2560	852	3.39	1.18	1.71
152	F	40s	37.8	Body aches	970	1.981	20	22	2664	1397	1.98	2.03	2.54
153	M	70s	39.3	Chest tightness	780	7.872	21	30	2984	NA	2.30	1.28	3.39
154	F	60s	37.7	Cough, body aches	920	1.451	18	22	3002	947	2.43	1.48	1.03
155	M	40s	38.5	Cough, fatigue	790	5.419	10	13	3112	2970	1.44	0.95	2.54
156	M	70s	37.5	Arthralgia, fatigue	720	3.830	18	28	3260	6900	1.50	0.94	2.07
157	M	50s	38.1	Cough, sputum	1510	1.309	19	23	3372	3168	4.28	3.91	2.46
158	F	50s	38	Chills	1000	0.933	21	33	3476	1133	2.62	1.19	2.54
159	F	60s	38	Cough	540	4.257	22	31	3502	NA	2.77	3.00	2.80
160	M	70s	38.5	Fatigue, muscle pain	730	0.476	16	22	3567	1900	2.88	2.62	4.60
161	M	50s	39.1	Cough, sore throat	750	0.675	26	32	3574	NA	3.14	2.50	4.03
162	M	30s	40	Cough, headache	680	6.347	25	29	3717	1835	3.56	2.96	3.52
163	F	80s	38.1	Cough, sore throat	560	1.712	17	26	3800	NA	1.92	1.46	1.47
164	F	50s	38	Fever	760	0.944	24	27	3830	NA	1.75	1.42	2.54
165	F	40s	37.6	Cough	1140	0.156	11	18	3831	5379	0.78	1.01	1.49
166	M	60s	39	Cough, fatigue	520	4.614	29	32	3977	6999	1.62	1.73	3.76
167	M	40s	37.6	Sore throat, fever, cough	760	3.628	22	30	4353	3592	3.14	1.55	4.22
168	F	50s	37.3	Fever	800	0.190	16	21	4534	5615	1.74	1.12	1.58
169	M	60s	38.6	Fever	870	1.057	10	24	4818	2972	0.74	0.91	1.22

170	M	60s	37.7	Fever	820	6.170	23	23	5121	5014	4.33	3.53	1.66
171	F	60s	39	Fever	590	4.460	17	18	6389	2560	2.86	1.31	3.68
172	F	60s	38	Fever, chills	1260	4.859	14	18	6552	4937	3.69	1.78	1.33
173	M	40s	38.5	Fever, muscle ache	1330	0.990	17	21	6888	5622	2.71	2.32	2.72
174	M	60s	38.5	Cough, shortness of breath, nausea	540	8.305	14	18	15989	NA	4.33	3.53	4.13
175	M	60s	37.8	Cough, shortness of breath	960	2.329	19	21	21567	38995	3.80	1.84	2.97

Abbreviations: CRP, C-reactive protein; ID50, 50% inhibitory dose; NA, not available; NAb, neutralizing antibody; OD 405, optical density at 405 nm; RBD, receptor-binding domain; S1, S2, spike proteins 1 and 2; TLC, total lymphocyte count.

SI conversion factors: To convert CRP to milligrams per liter, multiply by 10; and lymphocytes to $\times 10^9$ per liter, multiply by 0.001.

[a]The reference range for TLC is 1100/μL to 3200/μL.

[b]The reference range for blood CRP level is 0 to 1 mg/dL.

[c]Disease duration was defined as from the day of symptom onset to the day of discharge.

[d]Binding antibodies were tested at discharge.

eTable 2. Clinical characteristics of COVID-19 recovered patients with low, medium- low, medium-high, and high titers of SARS-CoV-2-specific NAbs on day of discharge and revisit

Time Point	Patient Information	Low	Medium-low	Medium-high	High
		<500	500-999	1000-2500	>2500
Discharge	Recovered Patient No. (%)	52 (30%)	29 (17%)	69 (39%)	25 (14%)
	Male (%)	19 (23%)	13 (16%)	36 (44%)	14 (17%)
	Female (%)	33 (35%)	16 (17%)	33 (35%)	11 (12%)
	Median Age (Years) [IQR]	38 [31-53]	42 [35-66.5]	56 [42-63.5]	63 [44-68]
	Hosp. Stay (Days) [IQR]	14.5 [12-19.5]	15 [13-20.5]	16 [12.5-21]	18 [15-22]
	Dx duration (Days) [IQR]	20 [16-24]	21 [16.5-26.5]	22 [18-25]	23 [21-29.5]
	Median NT (ID50)	327 [189-404]	715 [571-881]	1642 [1282-2090]	3800 [3316-4970]
Revisit	Recovered Patient No. (%)	39 (33%)	24 (21%)	35 (30%)	19 (16%)
	Male (%)	13 (23%)	13 (23%)	16 (28%)	14 (25%)
	Female (%)	26 (42 %)	11 (18%)	19 (31%)	5 (8%)
	Median Age (Years) [IQR]	40 [34-58]	42.5 [35.5-62]	52 [42-66]	58 [46-66]
	Hosp. stay (Days) [IQR]	14[12-17]	16 [13-17]	16 [12-21]	17 [11-23]
	Dx duration (Days) [IQR]	19 [15-23]	22 [19-24]	21 [17-26]	21 [18-28]
	Median NT (ID50) [IQR]	212 [114-379]	766 [638-862]	1373 [1133-1732]	3922 [2972-5615]

Abbreviations: IQR, interquartile range; Hosp., hospital; Dx, disease; NT, Neutralizing titer.

eTable 3. Clinical characteristics and SARS-CoV-2–specific NAb titers of younger, middle-aged, and older COVID-19 recovered patients.

**Patient Information**	**Age Distribution (Years)**		
	**15-39**	**40-59**	**60-85**
Recovered Patient No. (%)	55 (31)	64 (37)	56 (32)
Male (%)	27 (33)	33 (40)	22 (27)
Female (%)	28 (30)	31 (33)	34 (37)
Hosp. stay (days) [IQR]	14.5 [13-20]	16 [11-21]	17 [14-22]
Disease duration (days) [IQR]	21 [16-24]	21 [17-26]	22 [18-26]
Median NT (ID50) [IQR]	459 [255-998]	1291 [504-2126]	1537 [877-2427]

Abbreviations: IQR, interquartile range; Hosp., hospital; Dx, disease; NT, Neutralizing titer.

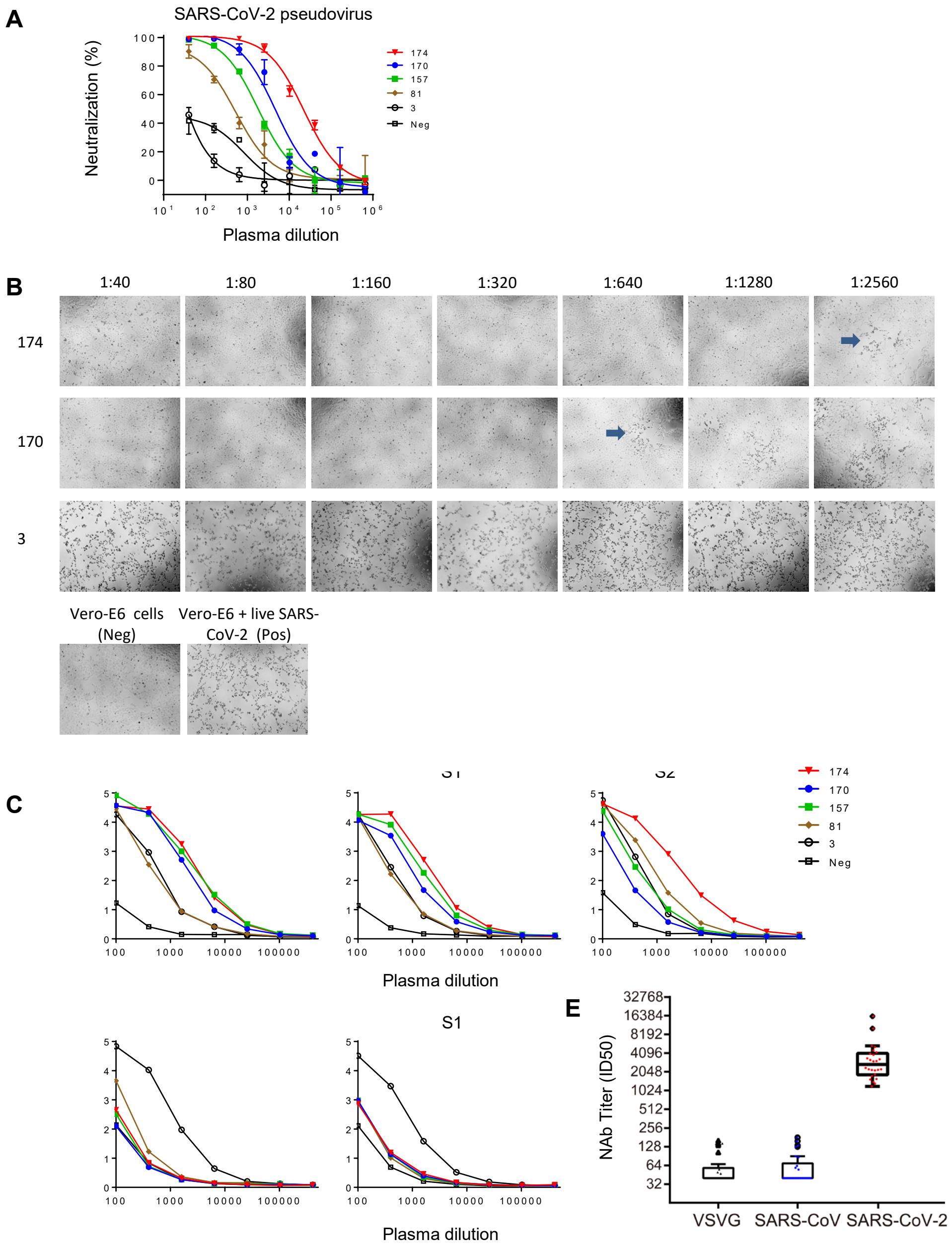

© 2020 Wu F et al. *JAMA Internal Medicine*.

eFigure 1. Plasma from patients who recovered from COVID-19 specifically inhibited SARS-CoV-2 infection but not SARS-CoV. (A) Plasma from five patients who recovered from COVID-19 inhibited infection of SARS-CoV-2. Plasma from a healthy donor was used as a negative control. The assay was performed in duplicate and the median percentage of neutralization is shown. (B) Inhibition of live SARS-CoV-2 infection by serial-diluted plasma of patient 3, 170 and 174. Vero-E6 cell was used as a negative control and cells infected by the live SARS-CoV-2 was used as a positive control. The viral cytopathology is indicated by arrows. (C) Binding of plasma from patients who recovered from COVID-19 to SARS-CoV-2 RBD, S1, and S2 proteins and (D) SARS-CoV RBD and S1 proteins. (E) NAb titers (ID50) of 26 plasma samples from patients who recovered from COVID-19 against VSV, SARS-CoV, and SARS-CoV-2 pseudovirus are shown as boxplots and scatter plots. Median NAb titier against SARS-CoV-2 (2685 [1826-4060]) is significantly higher than those against SARS-CoV (40 [40-69], median difference 2645 [95% CI 2132-3245], P<.001) and VSV-G negative control (40 [40-58], median difference 2645 [95% CI 2137-3236], P<.001). No significant difference is observed between NAb titers against SARS-CoV and VSV-G control (median difference 0 [95% CI 0-0], P=.96)

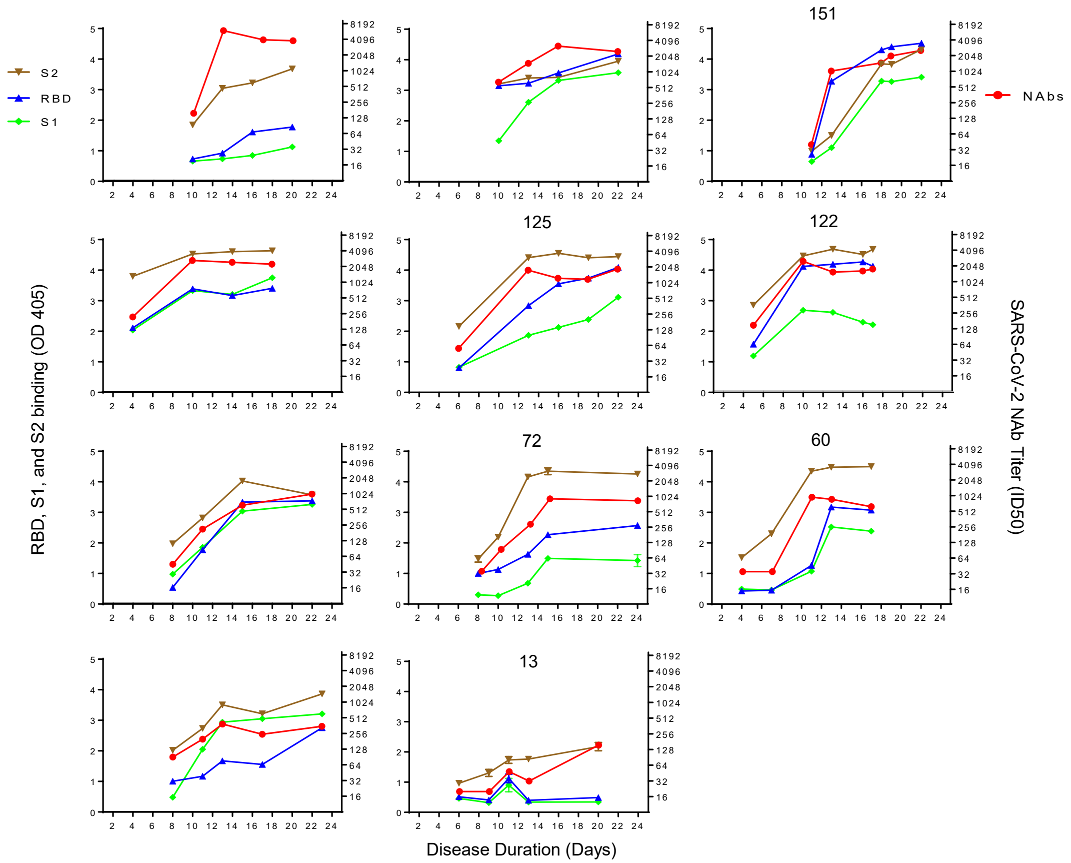

S2
RBD
S1
NAbs
151
125
122
72
60
13
RBD, S1, and S2 binding (OD 405)
SARS-CoV-2 NAb Titer (ID50)
Disease Duration (Days)

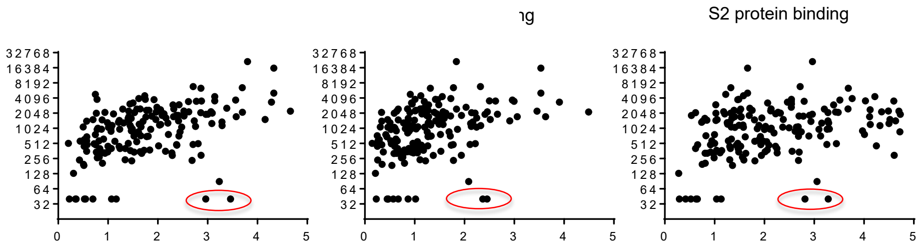

S2 protein binding

eFigure 2. Correlation between SARS-CoV-2 NAb titers and spike-binding antibodies. (A) The alignment of kinetics between SARS-CoV-2 NAb titers and binding antibodies to S1, RBD, and S2 for individual patient. (B) The correlations between SARS-CoV-2 NAb titers and binding antibodies to S1, RBD, and S2 in 175 patients at the time of discharge are shown. NAb titers among these patients positively correlate with the spike-binding antibodies targeting S1(r=0.451 [95% CI 0.320-0.564], $P<.001$), RBD (r=0.484 [95% CI 0.358-0.592], $P<.001$), and S2 regions (r=0.346 [95% CI 0.204-0.473], $P<.001$). Plasma from patient 3 and 8, which could not neutralize pseudovirus infection (ID50: <40) but developed high titers of spike-binding antibodies, are in red circle.

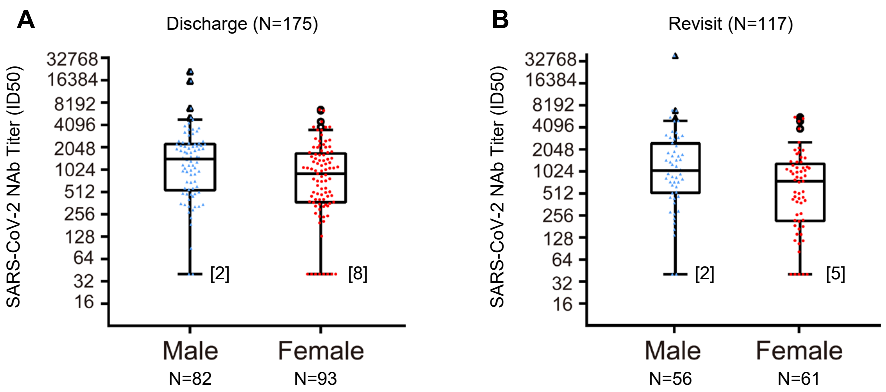
A
Discharge (N=175)
SARS-CoV-2 NAb Titer (ID50)
32768
16384
8192
4096
2048
1024
512
256
128
64
32
16
[2]
[8]
Male
N=82
Female
N=93
B
Revisit (N=117)
SARS-CoV-2 NAb Titer (ID50)
[2]
[5]
Male
N=56
Female
N=61

eFigure 3. Male patients who recovered from COVID-19 developed significantly higher NAb titers than females. SARS-CoV-2-specific NAb titers in male (blue) and female (red) recovered patients on the day of discharge (A) or revisit after two weeks (B) are shown as boxplots and scatter plots. NAb titers are significantly higher in male patients (1417 [541-2253], N=82) than those in females at the time of discharge (905 [371-1687], N=93, median difference 512 [95% CI 82-688], P=.011) and at follow-up (1049 [552-2454, N=56] vs 751 [216-1301, N=61], median difference 298 [95% CI 86-732], P=.009).

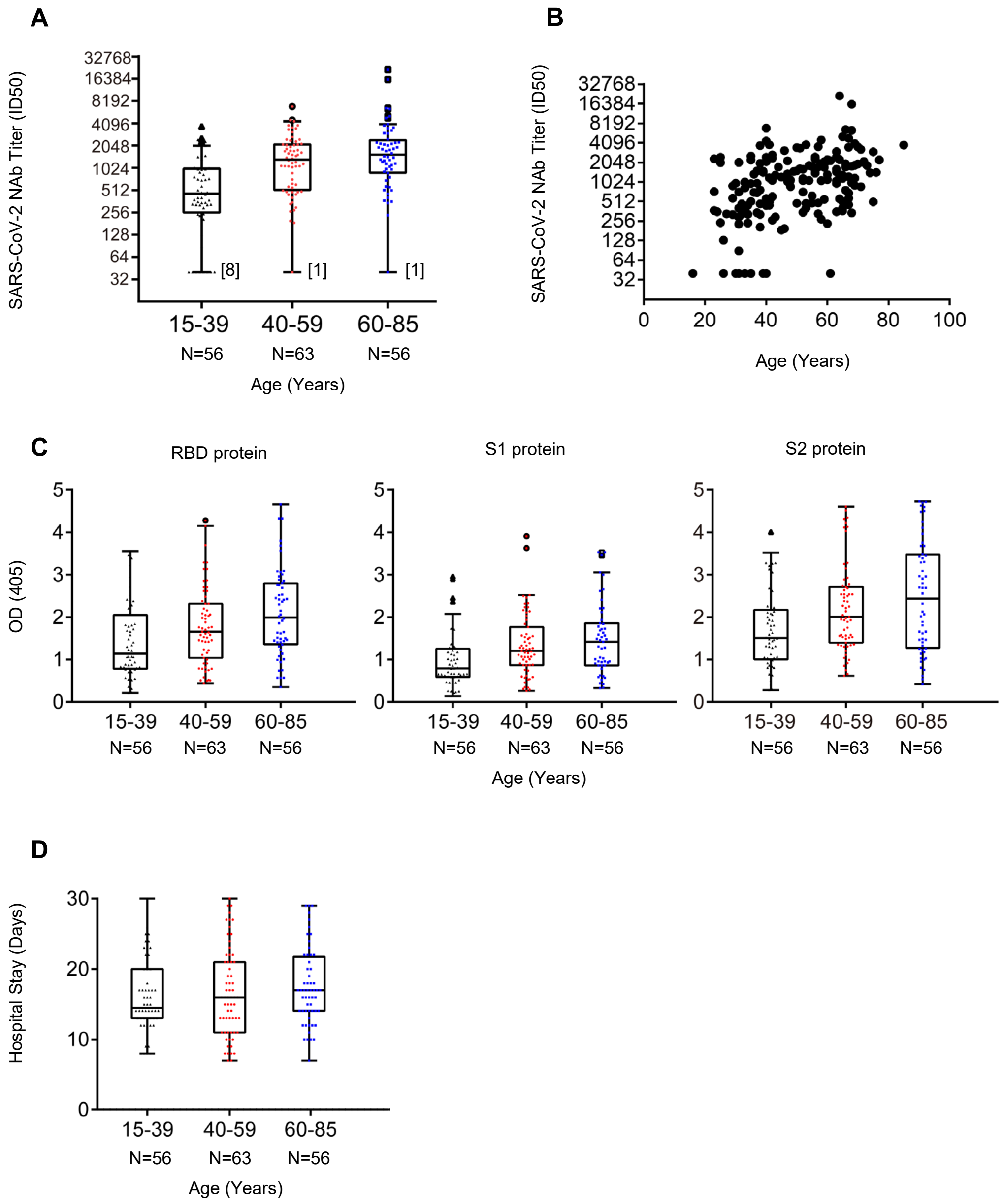

© 2020 Wu F et al. *JAMA Internal Medicine*. 18

eFigure 4. Older and middle-aged patients who recovered from COVID-19 developed higher levels of SARS-CoV-2–specific NAbs than younger recovered patients. (A) NAb titers of younger (15-39 years, N=56), middle-aged (40-59 years, N=63), and older (60-85 years, N=56) patients at time of discharge are shown as boxplots and scatter plots. Older (1537 [877-2427]) and middle-aged patients (1291 [504-2126]) had significantly higher plasma NAb titers than younger patients (459 [225-998] median difference 1078 [95% CI 548-1287], P<.001 and median difference 832 [95% CI 284-1013], P<.001). (B) Correlation between ages of patients and SARS-CoV-2-specific NAb titers at time of discharge (r=0.414 [95% CI 0.279-0.533], P<.001). (C) RBD, S1, or S2 binding antibodies levels of older (blue, N=56), middle-aged (red, N=63), and younger patients (black, N=56) who recovered from COVID-19 were compared. **RBD:** older OD 405 [IQR], 1.995 [1.365-2.8], median difference 0.885 [95% CI 0.31-1.01], p<.001 and middle-aged 1.66 [1.04-2.32], median difference 0.52 [95% CI 0.03-0.7], P=.03, vs younger 1.14 [0.783-2.05]); **S1:**older (1.44 [0.872-1.92], median difference 0.645 [95% CI 0.23-0.75], p<.001 and middle-aged 1.2 [0.87-1.77], median difference 0.405 [95% CI 0.13-0.58], P=.002 vs younger 0.795 [0.592-1.258]); **S2** older (2.44 [1.278-3.473], median difference 0.93 [95% CI 0.23-1.21], p=.002 and middle-aged 2.01 [1.4-2.72], median difference 0.5 [95%CI 0.12-0.82], P=.006 vs younger 1.51 [1.008-2.178]). (D) Length of hospital stay of younger 14.5 [13-20], middle-aged 16 [11-21], and older patients 17 [14-21.75]. No statistically significant difference was observed among the groups, P=.299, Kruskal-Wallis test)

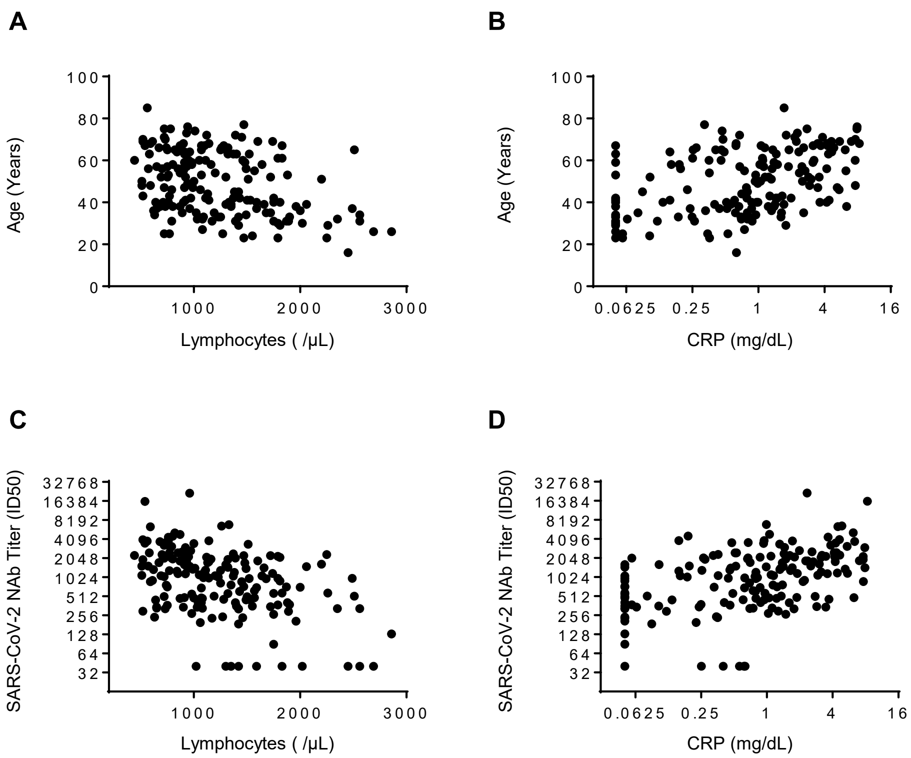
A
B
C
D
Age (Years)
Lymphocytes (/µL)
CRP (mg/dL)
SARS-CoV-2 NAb Titer (ID50)
0
20
40
60
80
100
1000
2000
3000
0.0625
0.25
1
4
16
32
64
128
256
512
1024
2048
4096
8192
16384
32768

eFigure 5. Correlation between SARS-CoV-2-specific NAb titers at discharge and lymphocyte count and CRP levels at admission. (A) Correlation between patient age and lymphocyte counts at admission (r= -0.355 [95% CI -0.482 - -0.214], p<.001). (B) Correlation between patient age and C-reactive protein (CRP) level at admission (r= 0.439 [95% CI 0.307-0.554], p<.001). (C) Correlation between SARS-CoV-2-specific NAb titers at discharge and lymphocyte counts at admission (r= -0.427 [95% CI -0.544 - -0.293], P<.001). (D) Correlation between SARS-CoV-2-specific NAb titers at discharge and CRP levels at admission (r=0.508 [95% CI 0.386-0.614], P<.001). Correlations were calculated by Spearman correlation test, N=175. The reference normal ranges are 1100-3200 /μL for lymphocyte counts and 0-1 mg/dL for blood CRP level.
